# Circ6401, a novel circular RNA, is implicated in repair of the damaged endometrium by Wharton’s jelly-derived mesenchymal stem cells through regulation of the miR-29b-1-5p/RAP1B axis

**DOI:** 10.1186/s13287-020-02027-5

**Published:** 2020-12-01

**Authors:** Qin Shi, Baolan Sun, Di Wang, Yi Zhu, Xinxin Zhao, Xiaoqing Yang, Yuquan Zhang

**Affiliations:** 1 Department of Obstetrics and Gynecology, Affiliated Hospital of Nantong University, 19 Xisi Road, Nantong, Jiangsu 226000 People’s Republic of China; 2 Department of Pediatrics, Affiliated Hospital of Nantong University, Nantong, People’s Republic of China; 3 Center for Reproductive Medicine, Affiliated Hospital of Nantong University, Nantong, People’s Republic of China; 4 Affiliated Maternity and Child Health Care Hospital of Nantong University, Nantong, People’s Republic of China

**Keywords:** hsa_circ_0006401, miR-29b-1-5p, Wharton’s jelly-derived mesenchymal stem cells, Endometrial stromal cells, Angiogenesis, RAP1B

## Abstract

**Background:**

Accumulating evidence indicates that mesenchymal stem cells (MSCs) exert tissue repair effects and therapeutic angiogenesis through their noncoding RNAs (ncRNAs). Our previous studies showed that MSCs derived from Wharton’s jelly (WJ-MSCs) can ameliorate damaged human endometrium by promoting angiogenesis. There is limited information on the functions and mechanism of ncRNAs in MSC-induced endometrial repair, and additional studies are needed for more insights.

**Methods:**

Here, WJ-MSCs were cocultured with or without endometrial stromal cells (ESCs) damaged by mifepristone (cocultured group versus non-cocultured group). TUNEL staining assays, EdU proliferation assays, flow cytometry apoptosis assays, and western blot assays were performed to observe the reparative effect of WJ-MSCs on damaged ESCs. Subsequently, circular RNA (circRNA) and microRNA microarrays were performed between the two groups. A subset of top upregulated circRNAs was validated by qRT-PCR. The functions of circ6401 (hsa_circ_0006401) in WJ-MSCs were investigated using lentivirus-mediated circRNA overexpression assays. The subcellular localization of circ6401 and miR-29b-1-5p in WJ-MSCs was identified by double RNA fluorescence in situ hybridization. Dual-luciferase reporter assays and western blot assays were performed to elucidate the regulatory mechanisms among circ6401, miR-29b-1-5p, and RAP1B.

**Results:**

WJ-MSCs significantly improved ESC proliferation and upregulated the expression of vascular angiogenesis markers. Circ6401 was upregulated in WJ-MSCs cocultured with damaged ESCs, while miR-29b-1-5p was significantly downregulated. Furthermore, circ6401 was found to bind to miR-29b-1-5p and prevent it from decreasing the level of RAP1B, a crucial protein involved in the VEGF signaling pathway, which promoted angiogenesis and stimulated the proliferation of ESCs.

**Conclusions:**

Our results showed the abundance and regulation profiles of ncRNAs of WJ-MSCs during repair of damaged ESCs and, for the first time, clarified the underlying mechanism by which circ6401 promotes endometrial repair by WJ-MSCs; thus, demonstrating that circ6401 may serve as a potential therapeutic target.

## Background

The endometrium plays a crucial role in embryo implantation and development. The integrity and continuity of the basal layer is the key to the periodic regeneration of the endometrium. Damage to the basal layer due to various reasons may cause endometrial aplasia or pathological alterations, resulting in amenorrhea, repeated abortion, and infertility [1].

Wharton’s jelly-derived mesenchymal stem cells (WJ-MSCs) exist in the Wharton’s jelly and perivascular area of the human umbilical cord and hold promise for the field of regenerative medicine because of their strong capacity to regenerate injured tissues indirectly through growth factor secretion and immunomodulation. In particular, several studies have found that transplantation of WJ-MSCs can significantly increase the number of microvessels at the injury site, improve local blood flow perfusion, accelerate wound healing, and promote organ function recovery [2–5]. Our previous studies showed that WJ-MSCs can ameliorate damaged human endometrial stromal cells (ESCs) [6, 7], thus indicating that it is possible to use WJ-MSCs to repair endometrial damage. To improve the efficiency of WJ-MSC treatment, it is necessary to further study the regulatory mechanism by which WJ-MSCs repair ESCs.

Recent studies have revealed that a large amount of endogenous noncoding RNAs (ncRNAs) exists in MSCs and that these ncRNAs have critical regulatory effects on cell metabolism, cell-to-cell communication, and immune regulation [8–10]. Circular RNA (circRNA) is a unique class of ncRNA that form a closed continuous loop by back-splicing with covalently joined 3′- and 5′-ends [11]. CircRNA molecules are rich in microRNA (miRNA) binding sites and can specifically bind miRNAs, thereby eliminating the inhibitory effect of miRNAs on target genes and upregulating their expression level, that is, functioning as competing endogenous RNA (ceRNA) [12]. A recent study showed that some circRNAs play an important role in gene regulation by acting as ceRNAs in MSCs. For instance, a circRNA originating from the FOXP1 gene (circFOXP1) acts as a miRNA sponge that targets miR-17-3p and miR-127-5p and promotes the proliferation and differentiation of MSCs [13]. The circRNA Circ_1983 participates in dicalcium silicate microparticle (C2S)-induced osteogenic differentiation of MSCs by sponge miR-6931 [14]. However, to date, little is known about the mechanisms of action of circRNAs in stem cell biology, especially in WJ-MSCs.

Rap1b, a highly homologous small G protein of Rap1a, is required for angiogenesis in vivo and for normal responses of endothelial cells (ECs) to vascular endothelial growth factor (VEGF). A recent study identified a novel mechanism of Rap1 in regulating responses of ECs to VEGF. Rap1 positively modulates VEGFR2 activation through the regulation of integrin α_v_β_3_ and works synergistically with VEGF signaling in vivo [15]. Our previous study showed that WJ-MSCs can reverse the damage of ESCs caused by mifepristone through upregulating the mRNA and protein expression of VEGF [6].

In the present study, we performed circRNA and miRNA microarray to detect differentially expressed ncRNA profiles in WJ-MSCs after coculturing with damaged ESCs. We found that circ6401 was upregulated, while miR-29b-1-5p was downregulated in the coculture group. Furthermore, circ6401 could bind to miR-29b-1-5p and prevent it from decreasing the level of RAP1B, a crucial protein involved in the VEGF signaling pathway, which promoted angiogenesis and stimulated the proliferation of ESCs.

## Methods

### Cell isolation and culture

Umbilical cords were collected from healthy women with full-term delivery. Endometrium samples were collected from patients with intramural or pedunculated subserosal hysteromyoma without endometrial abnormalities to obtain ESCs. None of the women had taken medication or had received hormonal therapy for at least 6 months before undergoing hysterectomy. The collection and use of human biological specimens were approved by the Ethics Committee of the Affiliated Hospital of Nantong University. WJ-MSCs and ESCs were isolated, cultured, and identified as previously described [16]. WJ-MSCs and ESCs were cultured in Dulbecco’s modified Eagle’s medium F12 (DMEM/F-12; Hyclone, USA) containing 10% fetal bovine serum (FBS; Gibco, USA), 100 U/ml penicillin, and 100 μg/ml streptomycin at 37 °C in a humidified 5% CO_2_ atmosphere.

### ESCs treated with mifepristone and cocultured with WJ-MSCs

The damaged ESC model and the WJ-MSC coculture system were developed according to our previous protocol [6]. Briefly, the ESCs were dispensed into 24-well plates at a density of 1.5 × 10^4^ cells/well and cultured in DMEM-F12 with 10% FBS. After 24 h, the medium was replaced with another medium containing 2% FBS. After 12 h, the cells were treated with mifepristone (#M8046; Sigma-Aldrich, USA) at the concentration of 60 μmol/L. The medium was then replaced with a fresh medium (DMEM-F12 with 2% FBS) after treatment for 48 h. The Transwell system (24 mm Transwell plate with a 0.4-μm pore polycarbonate membrane insert; #3412, Corning, NY, USA) was used to establish the coculture system. WJ-MSCs were seeded on top of the artificial basement membrane and placed in the upper part of the plate for coculture with damaged ESCs. The coculture was stopped after 48 h. ESCs were digested with 0.25% trypsin without ethylenediaminetetraacetic acid (EDTA) (Invitrogen, Carlsbad, CA, USA), and WJ-MSCs were digested with TrypLE Express (Thermo Fisher Scientific, USA).

### Deoxynucleotidyl transferase-mediated dUTP nick-end labeling (TUNEL) staining

Cells were fixed with 4% paraformaldehyde for 30 min at room temperature and washed twice with PBS. The cells were then incubated in the TUNEL solution containing FITC-dUTP for 60 min at 37 °C according to the manufacturer’s protocol (#C1088, Beyotime Biotechnology, China). Next, the cells were incubated in Hoechst 33342 for counterstaining of cell nuclei. Images were captured and analyzed using the EVOS M7000 Imaging System (Invitrogen, Thermo Fisher Scientific). The apoptosis rate was calculated as the ratio of FITC-positive cells (green cells) to total Hoechst 33342-positive cells (blue cells).

### 5-Ethynyl-2′-deoxyuridine (EdU) staining

DNA replication was detected by the EdU incorporation assay according to the manufacturer’s protocol (#C10310-3, RiboBio Co., China). Briefly, 1.5 × 10^5^ cells were inoculated in a 24-well plate. A labeling medium was added to each well for 2 h at 37 °C under 5% CO_2_. Cell samples were fixed with 4% paraformaldehyde for 30 min, permeated with 0.5% Triton X-100 for 10 min, and washed twice with PBS. The cell samples were then incubated with Pollo™ 488 dye buffer in caliginous condition for 30 min at room temperature. Subsequently, Hoechst 33342 was added, and the cell samples were incubated for 30 min. Images were captured and analyzed using the EVOS M7000 Imaging System. The EdU incorporation rate was calculated as the ratio of EdU-positive cells (green cells) to total Hoechst 33342-positive cells (blue cells).

### Cell apoptosis analysis by flow cytometry

ESCs from each group were collected and washed twice with cold PBS for apoptosis analysis. The cell apoptosis rate was determined using an annexin V-fluorescein isothiocyanate (FITC)/propidium iodide (PI) apoptosis detection kit (#KGA108, Keygen Biotechnology, China) according to the manufacturer’s instructions. Cells in each group were prepared in triplicate, and the stained cells were then sorted with a FACScan flow cytometer (Becton Dickinson, NJ, USA) within 1 h. The data were analyzed using Flow Jo software (Flow Jo, Ashland, OR, USA).

### Western blot assay

Total protein was extracted with RIPA buffer containing a protease inhibitor cocktail (Thermo Fisher Scientific). The protein concentration was determined using a Coomassie (Bradford) protein assay kit (Thermo Fisher Scientific). Equal amounts of total protein (30 μg) were separated by 12% SDS-PAGE and transferred onto PVDF membranes (Millipore, Billerica, MA, USA), followed by blocking with 5% skim milk. The membranes were incubated with antibodies against VEGFA (#ab46154, Abcam), VEGF Receptor 1 (#ab32152, Abcam), VEGF Receptor 2 (#ab194806, Abcam), RAP1B (#ab154756, Abcam), and GAPDH (#ab8245, Abcam) overnight at 4 °C. The membranes were then washed three times with TBST followed by incubation with secondary antibodies for 2 h at room temperature. Bands were detected by a chemiluminescence imaging system (Molecular Imager, ChemiDoc XRS+, Bio-Rad, USA) with Western Bright ECL HRP substrate (#WBKL0100, Millipore, USA). Band intensities were quantified using Image J software (NIH, Bethesda, MD, USA).

### RNA extraction and quantitative real-time PCR (qRT-PCR)

Total RNA containing small RNA was extracted using TRIzol (Invitrogen) according to the manufacturer’s instructions. RNA quality was analyzed with a NanoDrop 2000 spectrophotometer (Thermo Fisher Scientific, USA). To determine miRNA expression, reverse transcription was performed using miRNA-specific primers and a miScript reverse transcription kit (Qiagen, Germany) with RNU6B expression as an endogenous control. SYBR Green PCR Master Mix (Qiagen) was used to determine mRNA expression, with GAPDH expression as an endogenous control. To determine circRNA expression, cDNA reverse transcription and amplifications were conducted using a circRNA fluorescence quantitative detection kit (#GS0201-1, Geneseed Biotechnology, Guangzhou, China) according to the manufacturer’s instructions. qRT-PCR was performed using the ABI 7500 Real-Time PCR system (Applied Biosystems, USA). The 18s rRNA gene served as an endogenous control for normalization. Each relative RNA expression level was calculated from three different experiments. The sequences of gene-specific qRT-PCR primers are provided in Additional file 2: Table S1. The primers were designed using the Primer-BLAST program based on the National Center for Biotechnology Information (NCBI) sequence data. The relative expression level was calculated by the 2^−ΔΔCT^ method.

### circRNA and miRNA microarray analysis

CircRNA microarray analysis was performed with Human CircRNA Array v2 microarray (Capital Biotechnology, China). miRNA microarray analysis was performed with Affymetrix GeneChip miRNA 2.0 platform (Capital Biotechnology, China). Data were analyzed with GeneSpring software V13.0 (Agilent). The results of sample analysis are provided in Additional file 1: Fig. S1. To select differentially expressed genes, we used threshold values of ≥ 2 and ≤ − 2-fold change and a Benjamini-Hochberg corrected *p* value of 0.05. The data were log2 transformed and median centered by genes using the Adjust Data function of CLUSTER 3.0 software and then further analyzed with hierarchical clustering with average linkage. A comparative analysis between the T (cocultured WJ-MSCs) sample and N (non-cocultured WJ-MSCs) sample was performed using fold-change. Hierarchical cluster analysis was performed using complete linkage and Euclidean distance as a measure of similarity.

### circRNA-overexpressing plasmid construction and lentivirus infection

To generate WJ-MSCs with stable overexpressing circ6401, circ6401 cDNA was first synthesized and cloned into the pLC5-ciR vector (Geneseed) and confirmed by sequencing. Then, pLC5-circ6401 or pLC5-ciR empty vector was transfected into 293T cells by Lipofectamine 2000 (Invitrogen) to construct the lentivirus. The transfection efficiency was confirmed by qRT-PCR. After determining the viral titer, WJ-MSCs were infected by the obtained lentiviral particles.

### Prediction of circRNA-miRNA and miRNA-mRNA interactions

The interaction between circ6401 and miRNA was predicted using miRanda and Targetscan (http://www.microrna.org/microrna/ and http://www.targetscan.org/, respectively). The miRNA-mRNA interactions were predicted by Targetscan, while the miRNA binding sites were predicted by miRcode software (http://www.mircode.org/). Filtering restrictions were as follows: Context score ≥ 140.

### miRNA mimic or inhibitor transfection

All miRNA mimics, inhibitor, and their negative controls (miR-29b-1-5p mimics NC, miR-29b-1-5p inhibitor NC) were purchased from RiboBio and diluted to 50 nM. WJ-MSCs were transfected with miR-29b-1-5p inhibitor oligonucleotide, mimic oligonucleotide, or their negative controls by using Lipofectamine 2000 (Invitrogen), according to the manufacturer’s instructions. The transfection efficiency was confirmed by qRT-PCR.

### Double RNA fluorescence in situ hybridization (FISH)

Circ6401-overexpressing WJ-MSCs were transfected with miR-29b-1-5p mimics. After transfection, the cells were hybridized with a circ6401 probe (cy3-labeled) and an miR-29b-1-5p probe (FITC-labeled). Finally, the slides were stained with DAPI (Cell Signaling Technology, Boston, MA) and subjected to fluorescence signal detection under a Leica TCS SP8 laser confocal microscope (Leica). Sequences of FISH probes are shown in Additional file 2: Table S2.

### Dual-luciferase reporter assay

To evaluate the direct binding of miR-29b-1-5p to circ6401 or the 3′-UTR of RAP1B, the fragment of circ6401 or the 3′-UTR of RAP1B was first amplified by PCR and then cloned downstream of the Renilla gene in the psiCHECK-2 vector (Promega, Madison, WI, USA), which was named as circ6401-wt or RAP1B-wt, respectively. To generate the circ6401, RAP1B 3′-UTR mutant reporters, the seed regions of circ-001971, and the RAP1B 3′-UTR were mutated to remove the complementarity to miR-29b-1-5p, and the resulting constructs were named as circ6401-mut and RAP1B-mut, respectively. Cells were seeded into a 24-well plate. Next, miR-29b-1-5p mimics or NC was co-transfected with the wild-type vector or the mutant vector. Forty-eight hours after transfection, alterations in the luciferase activity were evaluated by the Dual-Luciferase Reporter Assay System (Promega) using firefly luciferase activity for normalization.

### Statistical analysis

Data were analyzed using GraphPad Prism software (GraphPad Software, La Jolla, CA, USA). Two-tailed Student’s *t* test was used to assess statistical significance in two groups, and one-way ANOVA with post hoc Bonferroni test was used in three or more groups. After the comparison test, the differences were considered significant at *p* < 0.05 or *p* < 0.001.

## Results

### Damaged ESCs were repaired by WJ-MSCs in vitro

**Fig. 1:**
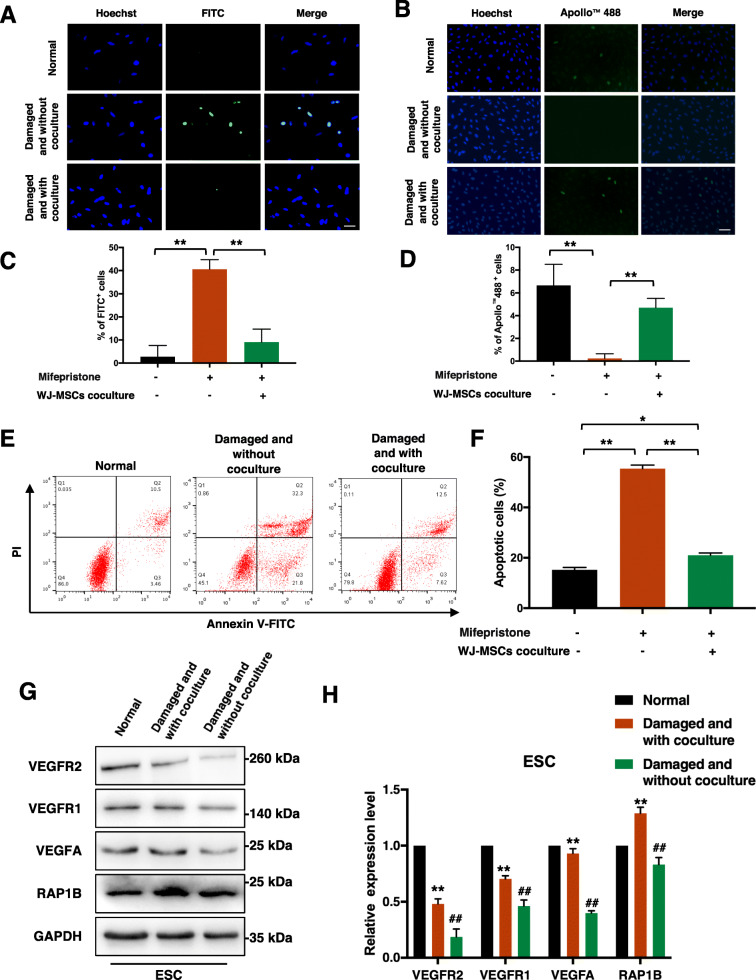
Damaged ESCs were repaired by WJ-MSCs in vitro*.* **a** TUNEL assay to detect apoptosis of ESCs in three groups. Scale bar, 20 μm. **b** EdU assay to detect proliferation of ESCs in three groups. Scale bar, 40 μm. **c**, **d** Quantitative analysis of TUNEL assay and EdU assay. Data in panel are shown as means ± standard deviation (*n* = 3). ^**^P < 0.001. **e**, **f** ESCs in three groups were stained with Annexin V-FITC and PI, and the percentage of apoptotic cells was detected by flow cytometry. Data in panel are shown as means ± standard deviation (*n* = 3). ^*^P < 0.05, ^**^P < 0.001. **g**, **h** VEGFA, VEGFR1, VEGFR2, and RAP1B protein expression of ESCs in three groups by western blot assay. Data in panel are shown as means ± standard deviation (*n* = 3). ^**^P < 0.01 compared with the damaged and without coculture group, ^##^*P* < 0.01 compared with the normal group

The TUNEL assay was performed first to confirm the apoptosis rate of ESCs in situ. Significant apoptosis was induced in ESCs after treatment with mifepristone for 48 h relative to that in the normal group (Fig. 1a). After coculturing with WJ-MSCs for 48 h, the apoptosis rate of the damaged ESCs decreased. Quantitative assessment revealed that the apoptosis rate in situ of ESCs in the coculture group was significantly lower than that in the non-coculture group (*p* < 0.001) (Fig. 1c). A subsequent EdU assay showed that the coculture with WJ-MSCs improved the proliferation of ESCs (Fig. 1b, d). In addition, the flow cytometry analysis showed that the apoptosis rate of ESCs in the coculture group was significantly lower than that in the non-coculture group (*p* < 0.001), but it was still higher than that in the normal group (*p* < 0.01) (Fig. 1e, f). We also detected protein changes in ESCs by western blot assay. The results showed that mifepristone treatment inhibited the expression of VEGFA, VEGFR1, VEGFR2, and RAP1B, while coculture with WJ-MSCs reversed the regulatory effects of mifepristone treatment on these vascular angiogenesis markers (Fig. 1g, h).

### WJ-MSCs show a variable circRNA expression profile when cocultured with damaged ESCs

**Fig. 2:**
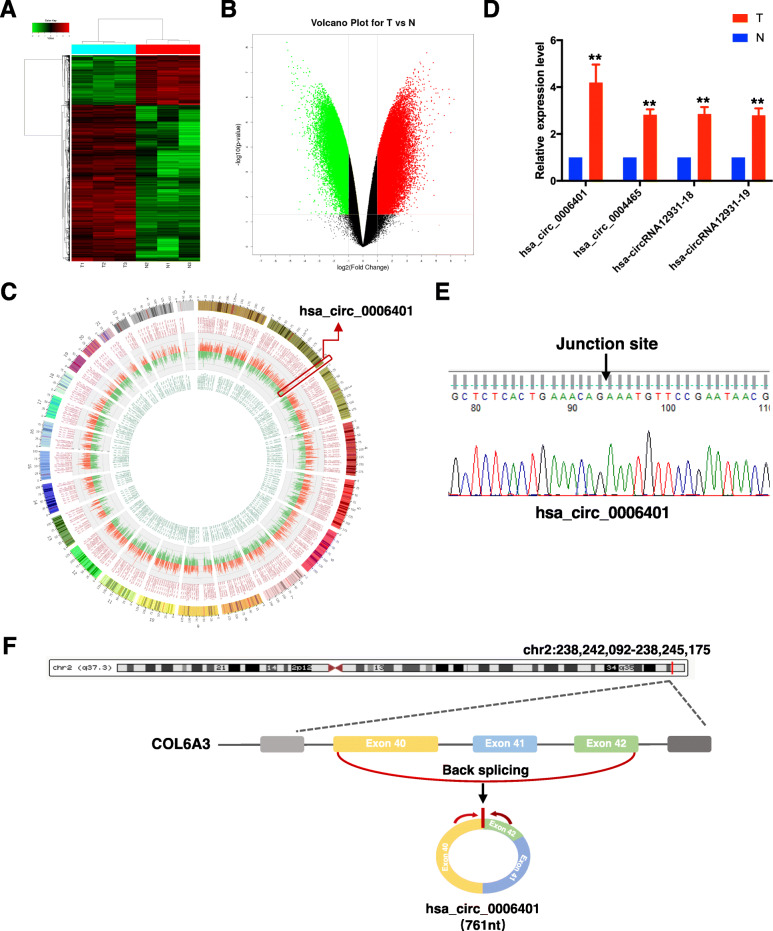
WJ-MSCs show a variable circRNA expression profile when cocultured with damaged ESCs. **a** The differences in circRNA expression profiles between the two groups by a circRNA microarray analysis are presented in the form of a heat map. N1–3, non-cocultured WJ-MSCs; T1–3, cocultured WJ-MSCs. Each group consisted of three individuals. CircRNAs are represented by single rows and samples by single columns. **b** Volcano plot of circRNA microarray analysis data. The values on the *X*-axes and *Y*-axes represent normalized fold changes and *P* values, respectively. The color scale indicates relative expression, upregulation (red) and downregulation (green). CircRNAs with fold change > 2 and *P* ≤ 0.05 were regarded as differentially expressed. **c** Circos plot shows the most significantly upregulated (red) and downregulated (green) circRNA expression profiles in the entire genome. **d** Four upregulated circRNAs’ expression level in WJ-MSCs by qRT-PCR. T, cocultured WJ-MSCs; N, non-cocultured WJ-MSCs. Data in panel are shown as means ± standard deviation (*n* = 3). ^**^P < 0.01 compare to non-coculture group. **e** Sanger sequencing of circ6401 qPCR amplification products. **f** Genomic location and splicing mode of circ6401

To determine whether circRNAs play a role in endometrium repair by WJ-MSCs, a circRNA microarray analysis of total RNA isolated from three pairs of cocultured and non-cocultured WJ-MSCs was performed. Principal component analysis (PCA) was performed to reflect the contribution rate of each principal component and the similarity between all samples. Sample analysis results are shown as a PCA plot, a box plot, and a correlation plot (Additional file 1: Fig. S1A-S1C). Differentially expressed circRNAs were selected for a clustering analysis by applying a fold-change filter based on *p* values ≤ 0.05. Among the detected circRNAs, 1551 showed differential expression, with a fold change ≥ 8. Of these 1551 circRNAs, 726 were upregulated and 825 were downregulated in cocultured WJ-MSCs. The differences in circRNA expression profiles between the two groups are presented as a heat map (Fig. 2a) and a volcano plot (Fig. 2b). Furthermore, the circos plot (Fig. 2c) shows the most significant circRNA expression profiles in the entire genome. Subsequently, a subset of top upregulated circRNAs was selected for further validation. The circRNAs that were consistent with the microarray data are shown in Fig. 2d. The presence of head-to-tail junction sequences was confirmed by RT-PCR and Sanger sequencing (Fig. 2e). qRT-PCR analysis showed that hsa_circ_0006401 (termed as circ6401), a circRNA originating from the COL6A3 gene (Fig. 2f), was the strongest regulated circRNA among the validated top upregulated circRNAs (Fig. 2d).

### circ6401 enhances the endometrium repair function of WJ-MSCs

**Fig. 3:**
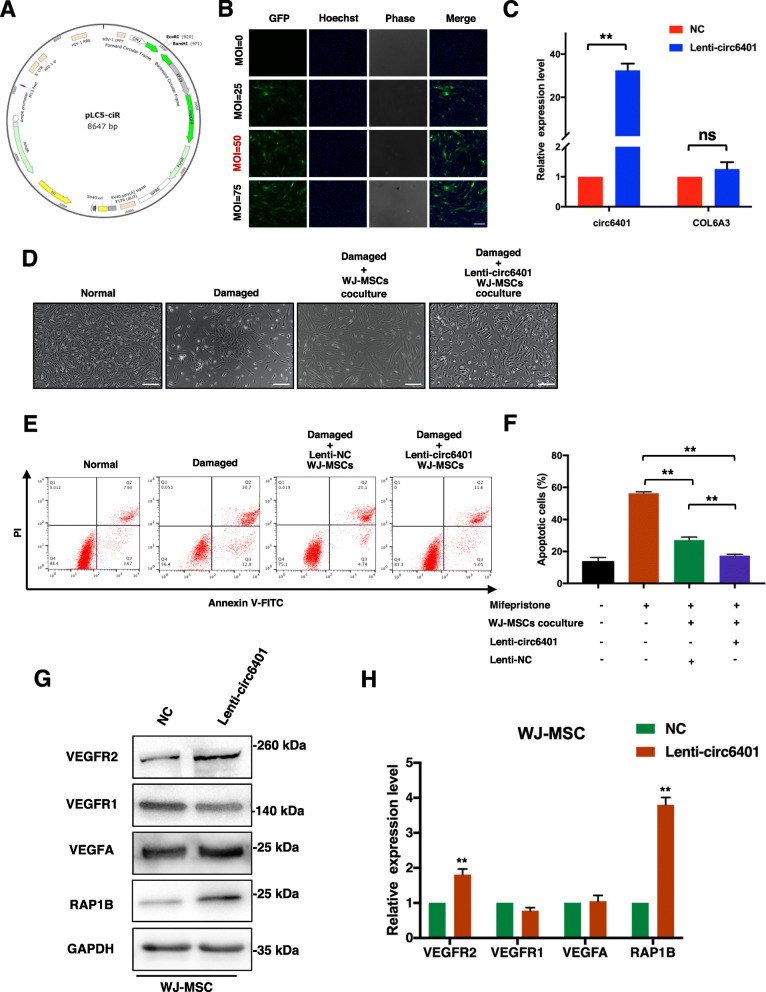
circ6401 enhances the endometrium repair function of WJ-MSCs. **a** Structure of the pLC5-ciR vector which contained a front and back circular frame. **b** GFP expression and infection efficiency were determined under fluorescence microscopy to select the optimal MOI for maximal transgene expression. MOI of 50 was finally used. Scale bar, 50 μm. **c** Efficiency of infected circ6401-overexpressing WJ-MSCs detected by qRT-PCR. ^**^P < 0.01. **d** The cell morphology of ESCs in the four groups. Scale bar, 500 μm. **e** Flow cytometry analysis was used to analyze ESC apoptosis after cocultured with lenti-circ6401^+^ WJ-MSCs or lenti-NC WJ-MSCs. **f** Quantitative analysis of flow cytometry analysis. Data in panel are shown as means ± standard deviation (*n* = 3). ^**^P < 0.01. **g** VEGFA, VEGFR1, VEGFR2, and RAP1B protein expression in lenti-circ6401^+^ WJ-MSCs, lenti-circ6401^−^ WJ-MSCs by western blot assay. **h** Quantitative analysis of western blot assay. Data in panel are shown as means ± standard deviation (*n* = 3). ^**^P < 0.01

To investigate the functions of circ6401 in WJ-MSCs, a circ6401-overexpressing plasmid was constructed (Fig. 3a). GFP expression and infection efficiency were determined by fluorescence microscopy to select the optimal multiplicity of infection (MOI) for maximal transgene expression (Fig. 3b). WJ-MSCs were finally infected with the circ6401 lentivirus (lenti-circ6401) at a MOI of 50. The results of qRT-PCR analysis showed that WJ-MSCs infected with lenti-circ6401 overexpressed circ6401 while and the parental gene of circ6401 was not changed (Fig. 3c). WJ-MSCs infected with lenti-circ6401 or lenti-NC were then used for coculturing with damaged ESCs. In the lenti-circ6401^+^ WJ-MSCs cocultured group, the cell morphology of ESCs was more similar to that in the normal group than those of ESCs in the lenti-NC WJ-MSCs cocultured group, and the number of nuclear vacuoles was lower than that in the damaged and non-cocultured group (Fig. 3d). The flow cytometry analysis showed that overexpression of circ6401 enhanced the effect of WJ-MSCs on decreasing the apoptosis rate of damaged ESCs (Fig. 3e, f). To validate whether circ6401 influences the expression of vascular angiogenesis markers, we next confirmed that overexpressing circ6401 increased the protein levels of VEGFR2 and RAP1B in WJ-MSCs, whereas it did not increase the protein levels of VEGFA and VEGFR1 (Fig. 3g, h).

### miR-29b-1-5p is downregulated during endometrium repair by WJ-MSCs and directly target 3′-UTR of RAP1B

**Fig. 4:**
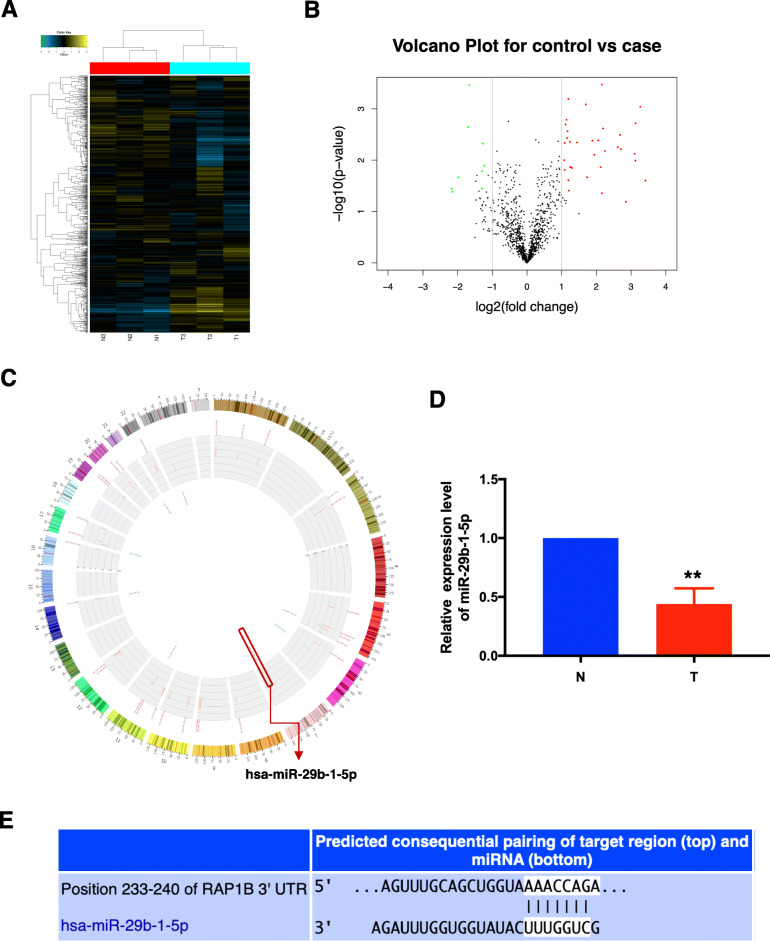
miR-29b-1-5p is downregulated during endometrium repair by WJ-MSCs. **a** The differences in miRNA expression profiles between the two groups by a miRNA microarray analysis are presented in the form of a heat map. N1–3, non-cocultured WJ-MSCs; T1–3, cocultured WJ-MSCs. Each group consisted of three individuals. miRNAs are represented by single rows and samples by single columns. **b** Volcano plot of miRNA microarray analysis data. The values on the *X*- and *Y*-axes represent normalized fold changes and *P* values, respectively. The color scale indicates relative expression, upregulation (red) and downregulation (green). miRNAs with fold change > 2 and *P* ≤ 0.05 were regarded as differentially expressed. **c** Circos plot shows the most significantly upregulated (red) and downregulated (green) miRNA expression profiles in the entire genome. **d** miR-29b-1-5p expression in WJ-MSCs cocultured with or without damaged ESCs by qRT-PCR. N, non-cocultured WJ-MSCs; T, cocultured WJ-MSCs. Data in panel are shown as means ± standard deviation (*n* = 3). ^**^P < 0.001. **e** The complementary pairing sequence between 3′UTR of RAP1B and miR-29b-1-5p

To investigate the molecular mechanisms through which circ6401 supports endometrium repair by WJ-MSCs, the expression of different miRNAs was detected by a miRNA microarray analysis. The results of sample analysis are shown as a PCA plot and a correlation plot (Additional file 1: Fig. S1D and S1E). Among the detected miRNAs, 43 miRNAs showed differential expression (fold change > 2.0, *p* values ≤ 0.05). Of these 43 miRNAs, 34 were upregulated and nine were downregulated in cocultured WJ-MSCs. The differences in miRNA expression profiles between the two groups are shown as a heat map (Fig. 4a) and a volcano plot (Fig. 4b). Furthermore, the circos plot (Fig. 4c) shows the most significantly upregulated (red) and downregulated (green) miRNA expression profiles in the entire genome. Interestingly, we found that the miRNA miR-29b-1-5p, which is significantly downregulated in cocultured WJ-MSCs as shown by qRT-PCR (Fig. 4d), may target 3′-UTR of RAP1B. The complementary pairing sequence between 3′-UTR of miRNAs and RAP1B mRNA was obtained from the TargetScan database (Fig. 4e). These results indicate that miR-29b-1-5p probably was regulated by circ6401 and play a role in the endometrium repair by targeting RAP1B.

**Fig. 5:**
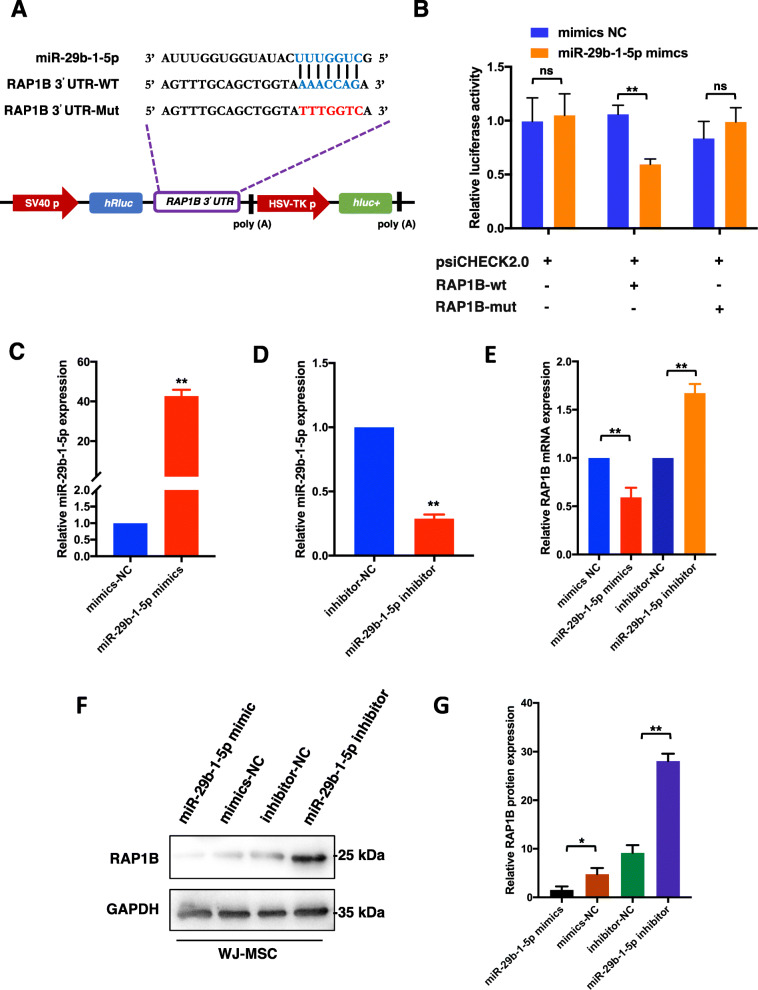
miR-29b-1-5p target 3′-UTR of RAP1B and regulates cellular function of WJ-MSCs. **a** 3′ UTR sequences containing the predicted “seed region” target site of RAP1B and its mut-sequences were cloned into the psiCHECK2.0 vector to prove whether RAP1B was the direct target gene of miR-29b-1-5p. **b** Dual-luciferase reporter gene assay to detect interaction between miR-29b-1-5p and RAP1B. RAP1B-mut, RAP1B 3′UTR mutant type; RAP1B-wt, RAP1B wild type; mimics NC, empty vector control. ^**^P < 0.001. **c**, **d** qRT-PCR was used to determine the miR-29b-1-5p expression level of WJ-MSCs transfected with mimics/inhibitor. ^**^P < 0.001 compared to mimics/inhibitor NC group. **e** qRT-PCR was used to determine the RAP1B mRNA expression level of WJ-MSCs in four groups. ^**^P < 0.001. **f** Western blot assay was performed to verify whether miR-29b-1-5p affected the RAP1B protein level of WJ-MSCs. **g** Quantitative analysis of western blot assay. Data in panel are shown as means ± standard deviation (*n* = 3). ^*^P < 0.05, ^**^P < 0.001

To prove whether RAP1B was the direct target gene of miR-29b-1-5p, 3′-UTR sequences containing the predicted “seed region” target site of RAP1B and its mut-sequences were cloned into the psiCHECK2.0 vector (Fig. 5a). The dual-luciferase reporter system showed significantly reduced luciferase activity in WJ-MSCs co-transfected with miR-29b-1-5p mimics and psiCHECK2.0-RAP1B-3′-UTR-wt (*p* < 0.001), whereas no significant reduction in luciferase activity was detected in those transfected with psiCHECK2.0 or co-transfected with miR-29b-1-5p mimics and psiCHECK2.0-RAP1B-3′-UTR-mut (Fig. 5b). These results clearly indicate that miR-29b-1-5p directly recognized and bound to the 3′-UTR of RAP1B.

To verify whether miR-29b-1-5p affected the RAP1B expression level, we transiently transfected WJ-MSCs with miR-29b-1-5p mimics/inhibitor and investigated the RAP1B mRNA and protein level at 24 and 48 h after transfection, respectively. The expression of miR-29b-1-5p was successfully upregulated and downregulated by the mimics and inhibitor, respectively (Fig. 5c, d). The qRT-PCR results showed that overexpression of miR-29b-1-5p dramatically suppressed the mRNA levels of RAP1B (*p* < 0.001), while knockdown of miR-29b-1-5p significantly improved the RAP1B expression level (*p* < 0.001) (Fig. 5e); these results were similar to western blot assay results (Fig. 5f, g). Taken together, these results showed that RAP1B is directly targeted and regulated by miR-29b-1-5p in WJ-MSCs.

### The circ6401/miR-29b-1-5p /RAP1B axis is involved in endometrium repair by WJ-MSCs

**Fig. 6:**
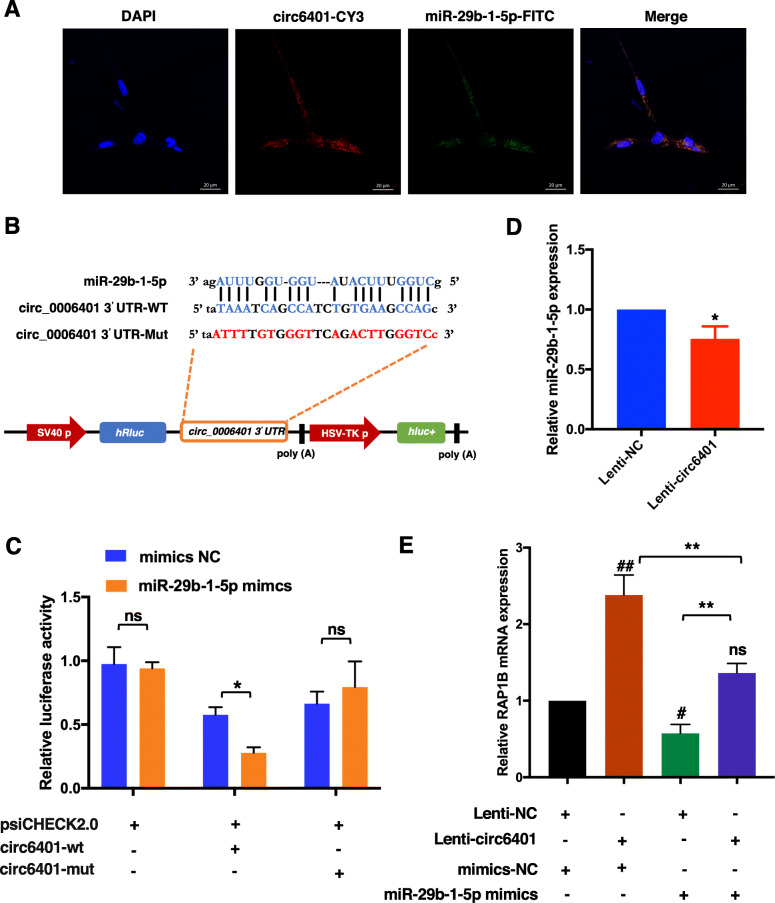
circ6401 acts as a sponge for miR-29b-1-5p and prevents it from inhibiting the translation of RAP1B in WJ-MSCs. **a** RNA FISH assay for subcellular co-localization of circ6401 and miR-29b-1-5p in WJ-MSCs. Cy3 labeled the circ6401 FISH probe, FITC labeled the miR-29b-1-5p FISH probe, and DAPI stained the cell nuclei. Scale bar, 20 μm. **b** 3′ UTR sequences containing the predicted “seed region” target site of circ6401 and its mut-sequences were cloned into the psiCHECK2.0 vector to prove whether circ6401 was the direct target gene of miR-29b-1-5p. **c** Dual-luciferase reporter gene assay to detect interaction between miR-29b-1-5p and circ6401. circ6401-mut, circ6401 3′UTR mutant; circ6401-wt, circ6401 wild type; mimics NC, empty vector control. ^*^P < 0.05. **d** qRT-PCR was used to determine miR-29b-1-5p expression level of WJ-MSCs transfected with lenti-NC/lenti-circ6401. Lenti-NC, lentivirus with empty vector; lenti-circ6401, lentivirus with circ6401 overexpressing vector. ^*^P < 0.05. **e** RAP1B mRNA expression in WJ-MSCs after co-transfection with circ6401 overexpression vectors and miR-29b-1-5p mimics. Data in panel are shown as means ± standard deviation (*n* = 3). ^**^P < 0.001. ^#^*P* < 0.05 compared to lenti/mimics NC group; ^##^*P* < 0.001 compared to lenti/mimics NC group; ^ns^*P* > 0.05 compared to lenti/mimics NC group

To establish whether circ6401 acts as a ceRNA by sequestering miRNAs present in the cytoplasm to inhibit the translation of their target mRNAs, we first performed RNA FISH assays to identify the subcellular localization of circ6401 and miR-29b-1-5p in WJ-MSCs. A Cy3-labeled probe specific for circ6401 and a FITC-labeled probe specific for miR-29b-1-5p were used for RNA FISH. The images indicate that both circ6401 and miR-29b-1-5p are mainly localized in a punctate pattern in the cytoplasm (Fig. 6a).

Next, the miR-29b-1-5p target site in the circ6401 sequence was mutated, and the dual-luciferase assay was repeated to verify the target relationships of miR-29b-1-5p and circ6401 (Fig. 6b). The results showed a significant effect on luciferase levels, demonstrating the specificity of the miR-29b-1-5p/circ6401 target site interaction (Fig. 6c). Moreover, the miR-29b-1-5p level was detected by qPCR, and lenti-circ6401^+^ WJ-MSCs showed lower miR-29b-1-5p level than lenti-NC WJ-MSCs (Fig. 6d).

Finally, WJ-MSCs with four types of treatments, namely NC group, circ6401 overexpression group, miR-29b-1-5p overexpression group, and co-overexpression of circ6401 and miR-29b-1-5p group, were used to assess the effects of circ6401/miR-29b-1-5p/RAP1B axis in WJ-MSCs. The qRT-PCR results again confirmed that miR-29b-1-5p overexpression significantly reduced RAP1B expression, which did not change even after co-overexpression of circ6401 and miR-29b-1-5p in WJ-MSCs, compared to the NC group (Fig. 6e).

## Discussion

In the past few years, the endometrial therapeutic effects of MSCs derived from different tissues such as bone marrow [17], umbilical cord [18, 19], human decidual [20], and uterus [21] have been investigated intensively, thus, providing new approaches to restore the endometrium to its normal morphology and function. In particular, the umbilical cord Wharton’s jelly was reported to be an ideal source of stem cells for clinical treatment and scientific research applications because of its highest concentration of allogeneic MSCs compared to that of other tissues [22]. Recently, ncRNAs have attracted much attention as fundamental players in the regulation of stem cell molecular networks. We previously reported the endometrial therapeutic effects of WJ-MSCs both in vitro [6] and in vivo [7]. Our recent study showed that 7757 circRNAs were differentially expressed in ESCs from the cocultured group [23]; however, the molecular mechanism by WJ-MSCs performing endometrial repair is still unclear, particularly with respect to ncRNAs. In the present study, for the first time, we determined the differentially expressed ncRNA profiles in WJ-MSCs after coculturing with damaged ESCs.

circ6401, a circRNA that was previously reported to participate in gastric cancer development by regulating miR-3064-5p-induced inhibitory effect on COL6A3 [24], has been rarely reported in MSCs and, thus, needed to be validated. In the present study, the circRNA microarray analysis and qPCR results demonstrated that circ6401 was most significantly upregulated in WJ-MSCs during coculture with damaged ESCs. The overexpression of circ6401 was then found to promote cell proliferation and increase the protein levels of VEGFR2 and RAP1B; this indicates that circ6401 exerted an angiogenesis role in WJ-MSCs-induced endometrial repair.

Many studies have revealed that MSCs exert tissue repair and therapeutic angiogenesis through their ncRNAs. For example, various cardioprotective exosomal miRNAs secreted by transplanted MSCs showed beneficial modulatory effects on the heart with myocardial infarction [25]. Kang et al. [26] found that adipose-derived stem cells induced angiogenesis through microvesicle transport of miRNA-31. Moreover, circRNA 100290 was reported to positively regulate angiogenesis induced by a conditioned medium of human amnion-derived MSCs through miR-449a/eNOS and miR-449a/VEGFA axes [27]. Previous studies have shown that circRNAs can function as miRNA sponges to competitively bind to miRNA, thus abrogating the inhibitory effect of miRNA on target genes [28–30]. Our data showed that miR-29b-1-5p, which was downregulated in WJ-MSCs during coculture with damaged ESCs, directly target 3′-UTR of RAP1B. Furthermore, the RNA FISH results indicated that both circ6401 and miR-29b-1-5p are mainly localized in the cytoplasm, thus allowing the circRNA-associated ceRNA regulatory pattern in WJ-MSCs. We further performed dual-luciferase assay and successfully verified the target relationships of circ6401, miR-29b-1-5p, and RAP1B. Our study provided first evidence that circ6401, a molecular decoy for miR-29b-1-5p, regulates RAP1B expression, thereby mediating the proliferation of ESCs.

Repressor/activator protein 1 (RAP1), a well-known shelterin protein, has been studied widely in cellular biology and cancers, and it regulates several basic cellular processes, including adhesion, polarity, and differentiation. Human RAP1 is specifically involved in protecting critically short telomeres and has important implications for the functions of telomeres in senescent cells [31]. In head and neck squamous cell carcinoma (HNSCC), RAP1 has been found to play a prominent role in cell-matrix adhesion through the extracellular matrix molecule fibronectin-induced α5β1 integrin and in cell migration through the RAP1/RAC1 signaling axis [32]. RAP1 is also involved in hematopoietic stem cell survival, oncogenesis, and response to chemotherapy, which was found to be independent of its association with the telomere or with its known partner TRF2 [33]. There are two Rap1 genes in vertebrates, which encode highly homologous Rap1a and Rap1b proteins. Lakshmikanthan et al. [34] indicated that RAP1 is a critical regulator of VEGFR2-mediated angiogenic and shear-stress EC responses, while RAP1B is the primary isoform essential for normal VEGF-induced EC barrier dissolution. In the present study, we showed that ESCs cocultured with WJ-MSCs expressed higher levels of VEGFR2 and RAP1B. Specifically, higher protein levels of VEGFR2 and RAP1B were detected in circ6401-overexpressing WJ-MSCs. Therefore, we hypothesized that a circRNA-RAP1B signaling axis was involved in the coculture system.

Recent increasing evidence has shown that several ncRNAs may target the RAP1B gene and regulate tumorigenesis, malignant progression, cell differentiation, etc. [35–39]. In colorectal cancer cells, RAP1B was found to be a direct and functional target of miR-30b-5p to regulate cell adhesion and mobility. However, the role of RAP1B in MSCs has been rarely studied. A previous study by Mendelson et al. [40] showed that megakaryocytes cocultured with MSCs had altered RAP1B gene expression and yielded platelets that are functional with low basal activation levels. An increased expression of RAP1B was observed in megakaryocytes cocultured with MSCs isolated from human umbilical cord arteries. Our results show that the RAP1B gene level was regulated through the circ6401/miR-29b-1-5p axis in WJ-MSCs, while both RAP1B and VEGFR2 protein levels were upregulated in the WJ-MSC/ESC coculture system and the proliferation of the damaged ESCs was enhanced. The results of the present research provide new insights into VEGF regulation by RAP1B in MSCs.

## Conclusions

In summary, our work describes for the first time the role of the circRNA circ6401 that binds to miR-29b-1-5p and prevents it from decreasing the level of RAP1B involved in the VEGF signaling pathway, which promoted angiogenesis and stimulated the proliferation of damaged ESCs. Acting as a potential therapeutic target, the circRNA circ6401 may play an important role in the regulation of endometrial repair by WJ-MSCs.

## Supplementary information

**Additional file 1: Fig. S1.** Sample analysis for the circRNA and miRNA microarray analysis. A, D Principal Component Analysis (PCA) 3D plot reflects the contribution rate of each principal component and the similarity between samples. B Box plot shows the overall gene (probe) expression before and after normalization of different samples for the circRNA microarray. C, E Correlation Plot reflects the similarity between samples. The value of each cell in the lower left is the correlation coefficient of the corresponding two samples. The color and area of the circle in the upper right indicate the degree of correlation of the corresponding sample.

**Additional file 2: Table S1.** Primer sequences for quantitative real-time PCR. Table S2. Sequences of FISH probes.

## Data Availability

The datasets used in this study are available from the corresponding author on reasonable request, and all generated data are included in the article and its additional files.

## Abbreviations

WJ-MSCs: Wharton’s jelly-derived mesenchymal stem cells
ESCs: Endometrial stromal cells
ncRNA: Noncoding RNA
circRNA: Circular RNA
miRNA: MicroRNA
VEGF: Vascular endothelial growth factor
RAP1: Repressor/activator protein 1
VEGFR: Vascular endothelial growth factor receptor
qRT-PCR: Quantitative real-time PCR
3′-UTR: 3′-Utranslated region
