# Supplementary material for: Circ6401, a novel circular RNA, is implicated in repair of the damaged endometrium by Wharton’s jelly-derived mesenchymal stem cells through regulation of the miR-29b-1-5p/RAP1B axis
Source: Stem Cell Res Ther. 2020 Dec 1;11:520. doi: 10.1186/s13287-020-02027-5 (PMC7708228; doi:10.1186/s13287-020-02027-5)
Supplement: Supplementary file 1 — Additional file 1: Fig. S1. Sample analysis for the circRNA and miRNA microarray analysis. A, D Principal Component Analysis (PCA) 3D plot reflects the contribution rate of each principal component and the similarity between samples. B Box plot shows the overall gene (probe) expression before and after normalization of different samples for the circRNA microarray. C, E Correlation Plot reflects the similarity between samples. The value of each cell in the lower left is the correlation coefficient of the corresponding two samples. The color and area of the circle in the upper right indicate the degree of correlation of the corresponding sample. [file 13287_2020_2027_MOESM1_ESM.pdf]

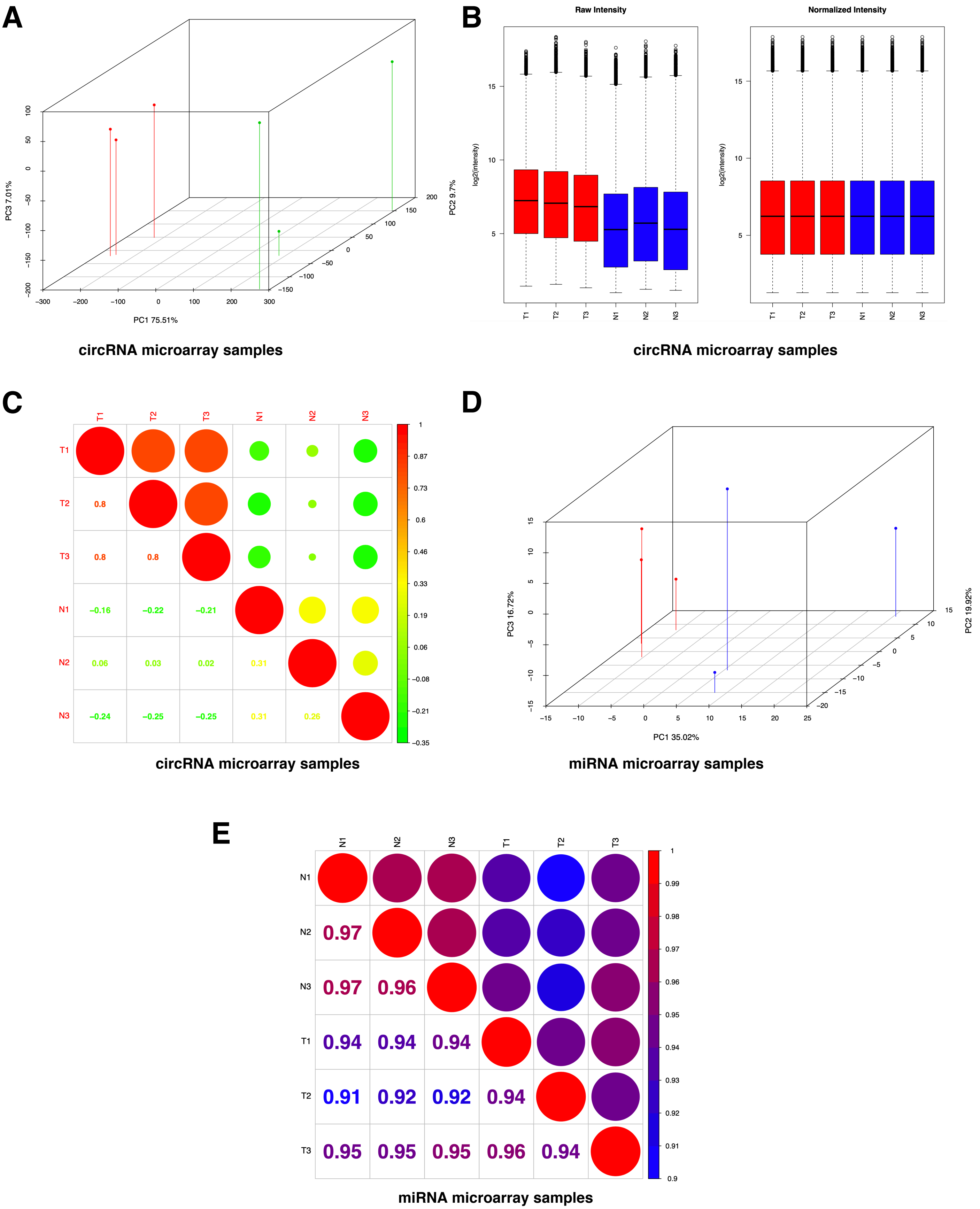
A
PC1 75.51%
PC3 7.01%
PC2 9.7%
circRNA microarray samples
B
Raw Intensity
Normalized Intensity
log2(intensity)
T1 T2 T3 N1 N2 N3
circRNA microarray samples
C
T1 T2 T3 N1 N2 N3
0.8
0.8 0.8
−0.16 −0.22 −0.21
0.06 0.03 0.02 0.31
−0.24 −0.25 −0.25 0.31 0.26
circRNA microarray samples
D
PC1 35.02%
PC3 16.72%
PC2 19.92%
miRNA microarray samples
E
N1 N2 N3 T1 T2 T3
0.97
0.97 0.96
0.94 0.94 0.94
0.91 0.92 0.92 0.94
0.95 0.95 0.95 0.96 0.94
miRNA microarray samples
