# Supplementary material for: Circ6401, a novel circular RNA, is implicated in repair of the damaged endometrium by Wharton’s jelly-derived mesenchymal stem cells through regulation of the miR-29b-1-5p/RAP1B axis
Source: Stem Cell Res Ther. 2020 Dec 1;11:520. doi: 10.1186/s13287-020-02027-5 (PMC7708228; doi:10.1186/s13287-020-02027-5)
Supplement: Supplementary file 2 — Additional file 2: Table S1. Primer sequences for quantitative real-time PCR. Table S2. Sequences of FISH probes. [file 13287_2020_2027_MOESM2_ESM.docx]

**Supplementary Tables**

**Table S1. Primer sequences for quantitative real-time PCR**

| **Gene** | **Primer direction** | **Sequence** |
| --- | --- | --- |
| GAPDH | Primer F | 5’-AGAAGGCTGGGGCTCATTTG-3’ |
|  | Primer R | 5’-GCAGGAGGCATTGCTGATGAT-3’ |
| COL6A3 | Primer F | 5'-AACCCTCCACATACTG CTAATTC-3' |
|  | Primer R | 5'-TCGTTGTCACTGGCT TCATT-3' |
| RAP1B | Primer F | 5’-GTGAATCCCTTGCTTGCTCAT-3’ |
|  | Primer R | 5'-AATACTGTGGCTCCCTGTTGG-3' |
| hsa_circ_0006401 | Primer F | 5'-GCTCTCACTGAAACAGAAATG-3' |
|  | Primer R | 5'-GGCTTCACAGATGGCTGATT-3' |
| hsa_circ_0004465 | Primer F | 5’-AGGTTTGCGCTCCTGATCAA-3' |
|  | Primer R | 5'-ATGGGTCGATGTTGCAGATG-3' |
| hsa_circRNA12931-18 | Primer F | 5'-TACCACGGAAGTTTCAGTACAA-3' |
|  | Primer R | 5'-GTAAAAAGCATTTTCACCTGTT-3' |
| hsa_circRNA12931-19 | Primer F | 5'-GGCTCTCACTGAAACAGATCA-3' |
|  | Primer R | 5'-CAACTGCCAAATCCACAGGAT-3' |
| hsa_miR-29b-1-5p | Primer F | 5'-GCTGGTTTCATATGGTGGTTTAGA-3' |

**Table S2. Sequences of FISH probes**

| **Gene symbol** | **Label** | **Probe sequence** |
| --- | --- | --- |
| hsa_circ_0006401 | CY3 | 5’-TTCGGAACATTTCTGTTTCAGTGAG-3’ |
| hsa-miR-29b-1-5p | FITC | 5’-TCTAAACCACCATATGAAACCAGC-3’ |
